# Comparative effectiveness of protein or protein-related supplementation combined with resistance or functional exercise for sarcopenia in older adults: a systematic review and network meta-analysis

**DOI:** 10.3389/fnut.2026.1892302

**Published:** 2026-08-25

**Authors:** Boran Yang, Xueyi Chen, Gaiping Yuan, Xinxin Zhang, Dongsheng Lu

**Affiliations:** Department of Exercise Science, School of Physical Education, Shaanxi Normal University, Xi’an, Shaanxi, China

**Keywords:** functional exercise, network meta-analysis, older adults, protein supplementation, resistance exercise, sarcopenia

## Abstract

**Background:**

Resistance and functional exercise are core non-pharmacological strategies for sarcopenia management. Protein or protein-related supplementation is commonly used to support exercise-induced adaptations, but the comparative effects of different supplementation strategies when added to exercise remain unclear.

**Objective:**

This systematic review and network meta-analysis compared the additional effects of different protein or protein-related supplementation strategies combined with resistance or functional exercise on grip strength, appendicular skeletal muscle mass index (ASMI), and timed up-and-go (TUG) performance in older adults with sarcopenia or at high risk of sarcopenia.

**Methods:**

PubMed, Embase, Cochrane Library, Web of Science, CNKI, and SinoMed were searched from inception to March 16, 2026. Randomized controlled trials comparing exercise plus protein or protein-related supplementation with the same or comparable exercise program plus placebo, no additional supplementation, or exercise alone were included. Risk of bias was assessed using the Cochrane Risk of Bias 2 (RoB 2) tool. Pairwise and frequentist network meta-analyses were conducted, and intervention rankings were assessed using the surface under the cumulative ranking curve (SUCRA).

**Results:**

Eighteen randomized controlled trials involving 1,341 participants and 12 combined intervention strategies were included. Pairwise meta-analysis showed that supplementation added to exercise significantly improved grip strength (SMD = 0.39, 95% CI: 0.20 to 0.58) and ASMI (SMD = 0.18, 95% CI: 0.00 to 0.35), but not timed up-and-go (TUG) performance (SMD = −0.39, 95% CI: −0.96 to 0.18). For TUG performance, negative SMD values indicate beneficial effects because lower TUG values represent better mobility. Network meta-analysis suggested potential outcome-specific effects of several supplementation strategies, including *β*-hydroxy-β-methylbutyrate (HMB)-based supplementation, whey- or leucine-enriched protein supplementation, and whey protein/essential amino acids combined with vitamin D strategies for grip strength, and whey- or leucine-enriched protein supplementation for ASMI. The combination of whey protein/essential amino acids and vitamin D with functional exercise showed a potential improvement in TUG performance; however, this finding was based on limited evidence and may largely reflect the contribution of functional exercise components. However, given the sparse evidence network and very low certainty of evidence, intervention rankings should be considered exploratory rather than definitive.

**Conclusion:**

Adding protein or protein-related supplementation to resistance or functional exercise may improve muscle strength and ASMI in older adults with sarcopenia or at high risk. HMB-, whey protein-, or leucine-based strategies showed potential benefits for specific outcomes; however, given the very low certainty of evidence and sparse comparative networks, no single supplementation strategy can be considered definitively superior.

**Systematic review registration:**

PROSPERO, CRD420261396976.

## 1 Introduction

Sarcopenia is a progressive and generalized skeletal muscle disorder that is highly prevalent among older adults, characterized by reduced muscle strength, low muscle quantity or quality, and impaired physical performance. The revised consensus of the European Working Group on Sarcopenia in Older People places low muscle strength at the center of case finding, using low muscle quantity or quality to confirm the diagnosis (1). Similarly, the 2019 update of the Asian Working Group for Sarcopenia emphasizes the combined assessment of muscle strength, muscle mass, and physical performance to identify sarcopenia in Asian populations (2). Given the association of sarcopenia with falls, frailty, disability, hospitalization, and mortality, effective non-pharmacological strategies are essential for preserving independence and promoting healthy aging (1, 3).

Resistance and function-oriented exercise, together with nutritional support, constitute the core non-pharmacological strategies for managing sarcopenia. Exercise-based interventions deliver the mechanical and neuromuscular stimuli required to maintain or improve muscle strength, mass, and physical performance. In parallel, protein or amino acid supplementation supplies substrates that may facilitate muscle remodeling. Expert recommendations advocate for adequate protein intake, particularly when combined with exercise, as an important component of optimizing muscle function in older adults (4, 5). Within this context, protein-related supplements such as whey protein, leucine, essential amino acids, and *β*-hydroxy-β-methylbutyrate (HMB) have attracted considerable attention. These nutrients may stimulate muscle protein synthesis, enhance anabolic signaling, or attenuate muscle protein breakdown (6).

Nevertheless, the incremental benefit of adding protein or protein-related supplementation to exercise-based interventions remains uncertain. Previous systematic reviews and recent meta-analytic evidence suggest that combined exercise and nutritional interventions may improve muscle-related outcomes in older adults. However, the magnitude of benefit varies considerably across populations, intervention contents, supplementation types, and outcome measures (7–10). Previous reviews, including recent network meta-analyses, have mainly focused on comparing different exercise–nutrition strategies or evaluating the overall effectiveness of combined interventions. For example, Liao et al. evaluated the comparative efficacy of different protein supplements combined with resistance training, primarily focusing on whether specific protein formulations provide differential benefits during exercise-based interventions. Similarly, Zhao et al. compared various exercise and nutrition strategies and evaluated their relative effectiveness for sarcopenia-related outcomes. However, these previous syntheses did not specifically address whether protein or protein-related supplementation provides additional benefits when an evidence-based exercise program is already implemented. Therefore, the present network meta-analysis specifically examined the incremental contribution of supplementation beyond comparable resistance or functional exercise programs. This distinction is clinically important because exercise is already considered a foundational intervention for sarcopenia management, and the practical clinical question is whether additional supplementation provides meaningful benefits beyond exercise alone and which supplementation strategy may be most appropriate for improving muscle strength, muscle mass, or physical performance.

Therefore, the present systematic review and network meta-analysis aimed to compare the additional effects of different protein or protein-related supplementation strategies when combined with resistance or functional exercise on grip strength, appendicular skeletal muscle mass index (ASMI), and timed up-and-go (TUG) test performance in older adults with sarcopenia or at high risk of sarcopenia.

## 2 Methods

The present systematic review and network meta-analysis were conducted in accordance with the Cochrane Handbook for Systematic Reviews of Interventions, the Preferred Reporting Items for Systematic Reviews and Meta-Analyses 2020 statement, and the PRISMA extension statement for network meta-analyses (11, 12). The review protocol was registered in the International Prospective Register of Systematic Reviews (PROSPERO registration number: CRD420261396976).

### 2.1 Data sources and search strategy

A systematic literature search was performed across PubMed, Embase, the Cochrane Library, Web of Science, China National Knowledge Infrastructure (CNKI), and SinoMed, covering database inception to March 16, 2026, without language restrictions. The search strategy combined controlled vocabulary terms and free-text keywords. Core search terms included “sarcopenia,” “muscle,” “resistance training,” “protein,” “amino acid,” “leucine,” “whey protein,” “*β*-hydroxy-β-methylbutyrate,” and “randomized controlled trial.” To identify potentially eligible studies missed by the electronic searches, the reference lists of included studies and relevant systematic reviews were manually screened.

### 2.2 Eligibility criteria

Studies meeting the following criteria were considered eligible. Participants were older adults aged 60 years or older with sarcopenia or at high risk of sarcopenia. Sarcopenia had to be diagnosed using recognized criteria, including the Asian Working Group for Sarcopenia (AWGS) criteria or the European Working Group on Sarcopenia in Older People (EWGSOP) criteria, as reported in the original studies (1, 2). High-risk populations were defined based on sarcopenia-related indicators, including reduced muscle mass, decreased muscle strength, or impaired physical performance, according to the criteria used in the original studies.

The experimental intervention was required to consist of resistance or function-oriented exercise combined with protein or protein-related supplementation. A function-oriented exercise was considered eligible when it included resistance, strengthening, balance, gait, chair-based, elastic-band, or body-weight components, provided that the exercise program was identical or comparable across groups. Protein-related supplementation included whey protein, milk protein, soy protein, leucine, essential amino acids, branched-chain amino acids, *β*-hydroxy-β-methylbutyrate, or multi-ingredient supplements containing protein- or amino-acid-related components. Eligible comparators comprised the same or comparable resistance- or function-oriented exercise program combined with placebo, no additional supplementation, or exercise alone. Thus, the present review focused on the additional effect of protein or protein-related supplementation when added to an exercise-based intervention, rather than comparing a combined exercise–nutrition intervention against no treatment.

Studies had to report at least one of the following outcomes: appendicular skeletal muscle mass index (ASMI), grip strength, or timed up-and-go (TUG) test. These outcomes were selected to represent key domains of sarcopenia management, including muscle mass, muscle strength, and physical performance. Although gait speed is commonly recommended for physical performance assessment in sarcopenia, it was not selected as a network meta-analysis outcome in the present study. Specifically, gait speed was reported in 11 eligible trials; however, the corresponding evidence network showed limited connectivity, with only one closed loop, and potential concerns regarding network consistency were identified during node-splitting analyses. Therefore, TUG was selected as the physical performance outcome because it was reported across a broader range of eligible comparisons, allowed quantitative synthesis within the available network, and provided a clinically relevant assessment of functional mobility. TUG was considered a complementary measure of physical performance and was not interpreted as equivalent to gait speed. To minimize bias and confounding and to ensure statistical reliability, only randomized controlled trials were included. Studies were excluded if they: (1) enrolled participants with sarcopenia accompanied by major comorbidities likely to influence muscle-related outcomes independently; (2) were case reports, descriptive studies, reviews, dissertations, or conference abstracts; (3) lacked full-text access or extractable outcome data; or (4) used protein intake only as part of the habitual diet rather than as additional supplementation. The complete eligibility criteria are presented in Supplementary Table S1.

### 2.3 Study selection

All retrieved records were imported into EndNote X9 for duplicate removal. Two reviewers independently screened titles and abstracts, and potentially eligible studies were subsequently assessed in full text. Disagreements during the screening process were resolved through discussion with a third reviewer. The study selection process is summarized in a PRISMA flow diagram.

### 2.4 Data extraction

Two reviewers independently extracted data using a predesigned extraction form. The extracted information included: (1) first author and publication year; (2) participant characteristics, including sample size, sex, and age; (3) sarcopenia diagnostic criteria and population status; (4) exercise modality, exercise intensity, intervention duration, protein or protein-related supplementation strategy, and comparator characteristics; (5) outcome measures and follow-up time points; and (6) outcome data.

When multiple time points were reported, data from the longest intervention duration were extracted to reduce selective reporting bias. When a single study included multiple eligible intervention arms, each eligible arm was extracted separately. For pairwise meta-analysis, shared control groups were split across relevant comparisons to avoid double-counting. For network meta-analysis, eligible arms were retained as separate comparisons within the corresponding network structure. Any discrepancies were resolved by discussion with a third reviewer.

Based on the extracted intervention characteristics, the intervention nodes were defined *a priori* according to clinically meaningful differences in supplementation composition and exercise characteristics. Alternative broader classifications (e.g., protein-based, leucine/HMB-based, dairy protein-based, or micronutrient-based interventions) were considered; however, they were not adopted because such aggregation could obscure potentially relevant differences in co-interventions, including vitamin D or omega-3 supplementation, as well as variations in exercise modality and training characteristics. Therefore, we retained the more specific node classification to preserve clinically interpretable intervention contrasts. Given the limited number of studies within several nodes, these comparisons should nevertheless be interpreted cautiously, and future network meta-analyses with larger evidence bases may further evaluate whether broader intervention categories improve statistical stability.

### 2.5 Risk-of-bias assessment

Methodological quality of the included randomized controlled trials was assessed using the Cochrane Risk of Bias 2.0 tool (13). The assessment covered five domains: (1) bias arising from the randomization process; (2) bias due to deviations from intended interventions; (3) bias due to missing outcome data; (4) bias in measurement of the outcome; and (5) bias in selection of the reported result. Each domain was rated as low risk, some concerns, or high risk. The overall judgment was classified as low risk when all domains were rated low risk; as some concerns when at least one domain was rated low risk and no domain was rated high risk; and as high risk when at least one domain was rated high risk or multiple domains had some concerns (14). Two reviewers independently performed the assessment, and disagreements were resolved by consensus or arbitration by a third reviewer.

### 2.6 Intervention node classification

For the network meta-analysis, intervention nodes were classified according to the primary supplementation component and the functional characteristics of the exercise program. Protein or protein-related supplements were grouped into nodes such as resistance training (RT) plus milk protein, RT plus soy protein, RT plus whey protein or leucine-enriched protein, RT plus HMB, resistance or balance/gait training plus whey protein/essential amino acids and vitamin D, and other multi-ingredient protein-related strategies.

When a supplement contained multiple ingredients, the node label reflected the dominant protein- or amino-acid-related component along with clinically relevant co-interventions, such as vitamin D or omega-3 fatty acids. This classification aimed to preserve clinically meaningful differences among supplementation strategies while maintaining sufficient network connectivity for comparative analysis. Detailed definitions of the intervention nodes for each outcome are provided in Supplementary Tables S2–S4.

### 2.7 Data synthesis and statistical analysis

All outcomes were continuous variables. Because measurement instruments and reporting approaches varied across studies, standardized mean differences (SMDs) with 95% confidence intervals (CIs) were used as effect estimates (15). For grip strength and ASMI, positive SMDs indicated beneficial effects. For TUG performance, negative SMDs indicated better physical performance, as lower values indicate greater mobility.

When studies did not directly report the mean and standard deviation of change scores, data were converted using published formulas (16). Given the anticipated methodological and clinical heterogeneity in exercise intensity, protein type, supplementation composition, and participant baseline characteristics, random-effects models were preferentially used for quantitative synthesis. Statistical heterogeneity was assessed using the *I*^2^ statistic and Cochran’s Q test; *I*^2^ ≥ 50% or *p* < 0.10 was considered indicative of substantial heterogeneity (17).

Pairwise meta-analyses were performed using Review Manager 5.4. When substantial heterogeneity was detected, prespecified subgroup analyses were conducted to explore potential sources. Subgroup variables included: (1) exercise intensity; (2) intervention duration; (3) participant status (confirmed sarcopenia or high-risk population); and (4) diagnostic criteria (AWGS, EWGSOP, or other criteria). If heterogeneity remained high within subgroups, random-effects models were used, and the findings were interpreted cautiously. Sensitivity analyses were conducted by excluding one eligible comparison at a time to examine the robustness of the pooled estimates and the influence of individual comparisons on overall heterogeneity (18). For multi-arm trials contributing more than one eligible comparison, sensitivity analyses were interpreted at the comparison level unless all eligible comparisons from the same trial were removed simultaneously. For outcomes with at least 10 studies and substantial heterogeneity, random-effects meta-regression using restricted maximum likelihood estimation was planned, with intervention duration, baseline ASMI, and mean daily protein intake as covariates (19).

Network meta-analysis was performed using Stata 15.1. Network evidence plots were generated to visualize the structure of direct and indirect comparisons, and network meta-analysis models were fitted within a frequentist framework using Stata’s network meta-analysis routines (20). Before model fitting, the transitivity assumption was assessed by comparing potential effect modifiers across intervention comparisons. Specifically, we evaluated whether important clinical and methodological characteristics were similarly distributed across treatment comparisons, including participant age, available sex distribution, sarcopenia diagnostic criteria, baseline population status (confirmed sarcopenia or high risk), exercise modality and intensity, intervention duration, supplementation characteristics (type and composition), and outcome measurement methods. Network consistency was assessed by comparing direct and indirect evidence using node-splitting analysis.

When closed loops were available, global and local inconsistency were assessed, including node-splitting analyses. If no statistically significant inconsistency was detected between direct and indirect evidence, a consistency model was used; otherwise, an inconsistency model was considered. The surface under the cumulative ranking curve (SUCRA) was used to rank interventions by relative effectiveness, with higher values indicating a greater probability of being among the most effective strategies. However, rankings were interpreted together with effect estimates, confidence intervals, network geometry, inconsistency assessment, and sensitivity analyses, rather than as independent evidence of superiority.

Sensitivity analyses for the network meta-analysis were conducted by excluding low-frequency or small-sample intervention nodes that appeared only once in the original network and refitting the model. For outcomes with at least 10 studies, comparison-adjusted funnel plots were used to assess potential publication bias and small-study effects (21).

### 2.8 Certainty of evidence

The certainty of evidence was assessed using the Grading of Recommendations Assessment, Development, and Evaluation (GRADE) approach (22–24). Evidence from randomized controlled trials was initially rated as high certainty and then downgraded according to risk of bias, inconsistency, indirectness, imprecision, and publication bias. For network meta-analysis estimates, the assessment considered the contribution of direct and indirect evidence, the coherence between direct and indirect comparisons, and the precision of the network estimates (23).

For the main outcomes and key comparisons contributing to the primary conclusions, the certainty of the evidence was independently assessed by two reviewers. Disagreements were resolved by discussion or consultation with a third reviewer.

## 3 Results

### 3.1 Study selection

**Figure 1:**
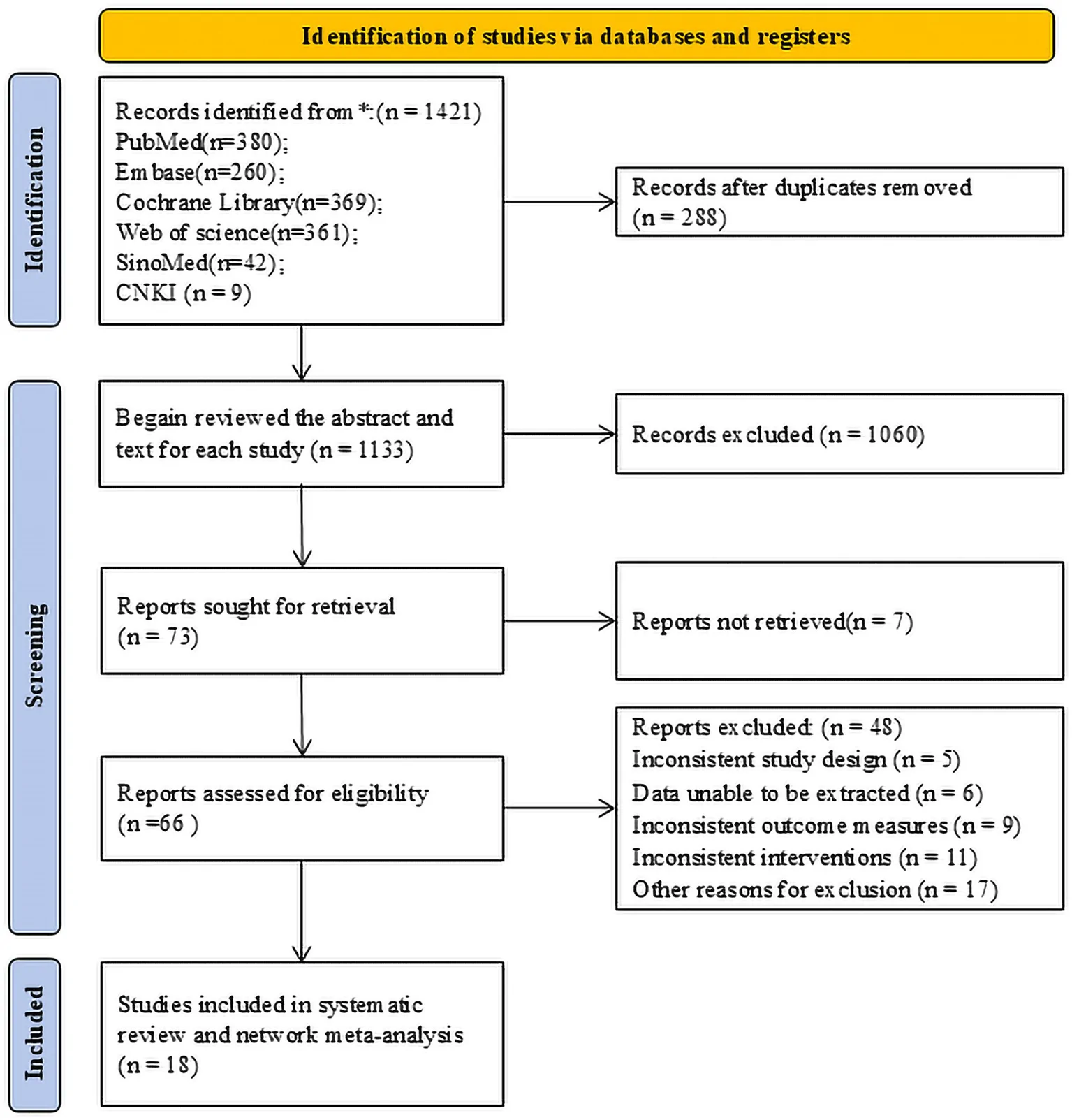
PRISMA flow diagram of study selection.

The initial database search yielded 1,421 records. After duplicate removal and title-and-abstract screening, 73 articles were assessed in full text. Following full-text review, 18 randomized controlled trials met the inclusion criteria for the systematic review and network meta-analysis (25–42). These trials evaluated protein or protein-related supplementation added to resistance- or function-oriented exercise in older adults with sarcopenia or at high risk of sarcopenia. Figure 1 presents the study selection process.

### 3.2 Characteristics of the included studies

A total of 18 randomized controlled trials involving 1,341 participants were included. Among these, three trials employed a multi-arm design (26, 41, 42), while the remaining studies were two-arm. Participants were older adults with confirmed sarcopenia or at high risk of sarcopenia. Sex distribution was reported inconsistently across the included trials. Although Table 1 summarizes the available male and female participant numbers reported by individual studies, an accurate overall sex breakdown could not be determined because several studies did not provide sex-specific information for the full randomized participants. The intervention arms encompassed 12 exercise-based protein or protein-related supplementation strategies. In contrast, comparator arms consisted of the same or a comparable exercise program combined with placebo, no additional supplementation, or exercise alone.

**Table 1:** Characteristics of the included studies and eligible intervention arms.

Author	Year	Sample size (n)	Sex (male/female, n)	Age (years)	Diagnostic criteria	Population status	Follow-up duration	Exercise intensity	Intervention	Outcomes
Li et al. (25)	2021	169	IG:22/26 CG:14/23	IG: 71.52 ± 5.28 CG: 73.73 ± 5.69	AWGS	Confirmed	12 weeks	High	ST + WP + FO/VD	①②
Maltais et al. (26)	2016	26	MG:8/0 SG:8/0 CG:10/0	MG:68 ± 5.6 SG:64 ± 4.8 CG:64 ± 4.5	Others	Confirmed	16 weeks	High	RT + MP RT + SP	②③
Mori and Tokuda (27)	2022	70	IG:3/23 CG:4/23	IG:77.7 ± 3.3 CG: 77.6 ± 5.2	AWGS	Confirmed	48 weeks (24-week intervention + 24-week detraining follow-up)	High	EBT + BW + WP	①②
Rondanelli et al. (28)	2020	127	IG: 26/38 CG: 17/46	IG: 81 ± 7 CG: 82 ± 5	EWGSOP	Confirmed	4–8 weeks	High	RBT + WP/EAA + VD	①②③
Yamada et al. (29)	2019	112	IG:8/20 CG:10/18	IG: 84.9 ± 5.6 CG: 84.7 ± 5.1	AWGS	Confirmed + at risk	12 weeks	Low	BW + Prot+VD	①
Rondanelli et al. (30)	2016	130	IG: 29/40 CG: 24/37	IG: 80.77 ± 6.29 CG: 80.21 ± 8.54	Others	Confirmed	12 weeks	High	RBT + WP/EAA + VD	①
Nilsson et al. (31)	2020	32	IG: 16/0 CG: 16/0	IG: 74.4 ± 1.3 CG: 77.4 ± 2.8	EWGSOP	Confirmed	12 weeks	Low	FBEB+MIS	①②
Molnar et al. (32)	2016	34	IG:7/10 CG: 5/12	IG:66.59 ± 1.63 CG: 66.35 ± 1.79	EWGSOP	Confirmed + at risk	3 months	Low	RBT + WP/EAA + VD	①
Lin et al. (33)	2024	40	IG: 12/8 CG: 11/9	IG: 76.06 ± 4.65 CG: 75.43 ± 4.88	Others	Confirmed	48 weeks	Low	RT + Prot(Whey/Leu)	①②
Osuka et al. (34)	2021	104	IG:0/36 CG:0/38	IG: 73.5 ± 4.2 CG: 71.8 ± 4.1	AWGS	At risk	12 weeks	High	RT + HMB	①③
Zhu et al. (35)	2019	76	IG: 7/29 CG: 11/29	IG: 74.8 ± 6.9 CG: 74.5 ± 7.1	AWGS	Confirmed	12 weeks	Low	CRT + Prot (HMB/VD/O3)	②
Meza-Valderrama et al. (36)	2024	32	IG: 3/14 CG: 5/10	IG: 81.8 ± 8.8 CG: 81.3 ± 10.2	EWGSOP	Confirmed	12 weeks	Low	RT + HMB	①
Takeuchi et al. (37)	2019	68	IG: 15/20 CG: 14/19	IG: 78.8 ± 5.1 CG: 80.9 ± 7.3	AWGS	Confirmed	8 weeks	Low	L-IRT + BCAA+VD	①
Tokuda and Mori (38)	2023	36	IG: 2/13 CG: 2/13	IG: 78.0 (77.0,79.0) CG: 79.0 (77.0, 80.3)	AWGS	Confirmed	24 weeks	High	RBT + AA	①
Yang et al. (39)	2023	34	IG: 7/11 CG: 5/11	IG:72.89 ± 7.02 CG: 71.44 ± 5.22	AWGS	Confirmed	12 weeks	Low	RT + HMB	①②
Vijayakumaran et al. (40)	2023	16	IG:0/8 CG:0/8	IG: 66.6 ± 4.0 CG:65.5 ± 1.9	AWGS	At risk	12 weeks	High	RT + MP	①②
Chiang et al. (41)	2021	35	MG:10/2 SG:9/2 CG:10/2	MG:84.9 ± 6.2 SG:84.9 ± 6.2 CG:84.9 ± 6.2	AWGS	Confirmed	12 weeks	Low	RT + MP RT + SP	①②
Roschel et al. (42)	2021	44	IG:0/22 CG:0/22	IG:72 ± 6 CG:72 ± 6	Others	At risk	16 weeks	High	RBT + AA	①③
Roschel et al. (42)	2021	66	IG:0/22 SG:0/22 CG:0/22	IG:72 ± 6 SG:72 ± 6 CG:72 ± 6	Others	At risk	16 weeks	High	RT + Prot(Whey/Leu) RT + SP	①③
Roschel et al. (42)	2021	44	IG:0/22 CG:0/22	IG:72 ± 6 CG:72 ± 6	Others	At risk	16 weeks	High	RT + Prot(Whey/Leu)	③
Roschel et al. (42)	2021	46	IG:23/0 CG:23/0	IG:72 ± 6 CG:72 ± 6	Others	At risk	16 weeks	High	RT + Prot(Whey/Leu)	③

AWGS, Asian Working Group for Sarcopenia; EWGSOP, European Working Group on Sarcopenia in Older People; ST+WP+FO/VD, strength training plus whey protein, fish oil, and vitamin D; RT+MP, resistance training plus milk protein; RT+SP, resistance training plus soy protein; EBT+BW+WP, elastic band training plus body-weight exercise and whey protein; RBT+WP/EAA+VD, resistance/balance/gait training plus whey protein/essential amino acids and vitamin D; BW+Prot+VD, body-weight exercise plus protein and vitamin D supplementation; FBEB+MIS, full-body elastic band exercise plus multi-ingredient supplementation (e.g., Muscle5); RT+Prot (Whey/Leu), resistance training plus protein supplementation (whey protein or leucine); RT+HMB, resistance exercise plus HMB or Ca-HMB; CRT+Prot (HMB/VD/O3), chair-based resistance training plus protein-related supplementation containing HMB, vitamin D, and omega-3 fatty acids; LIRT+BCAA+VD, low-intensity resistance training plus branched-chain amino acids and vitamin D; RBT+AA, resistance/elastic-band/balance and gait training plus amino acids such as EAA or leucine. ① grip strength; ② appendicular skeletal muscle mass index (ASMI); ③ timed up-and-go test (TUG); IG, intervention group; CG, control group; MG, milk-protein group; SG, soy-protein group. Age is reported as mean ± SD or median with range/interquartile range when applicable. Some multi-arm or multiple-comparison studies contributed more than one eligible comparison; therefore, the same study may appear in more than one row. Sex distribution represents the male and female participant numbers reported in the original studies. In some trials, sex-specific information was not available for the entire randomized sample; therefore, the sum of reported male and female participants may not always equal the total sample size.

Intervention duration ranged from 4 to 48 weeks. The included interventions varied considerably in exercise modality, exercise intensity, supplementation type, and supplementation composition. Supplementation strategies included whey protein, milk protein, soy protein, leucine-enriched protein, essential amino acids, branched-chain amino acids, HMB, and multi-ingredient protein-related supplements. Table 1 summarizes the main characteristics of the included studies and eligible intervention arms.

### 3.3 Risk-of-bias assessment

In the risk-of-bias assessment, seven studies raised some concerns in the randomization domain. Regarding deviations from intended interventions, one study was judged to be high risk, and another to have some concerns. For missing outcome data, two studies were rated as high risk, and one study was rated as having some concerns. For outcome measurement, two studies were judged to be high risk, and one study was judged to have some concerns. Regarding the selection of the reported results, seven studies were rated as high risk and 10 as some concerns.

**Figure 2:**
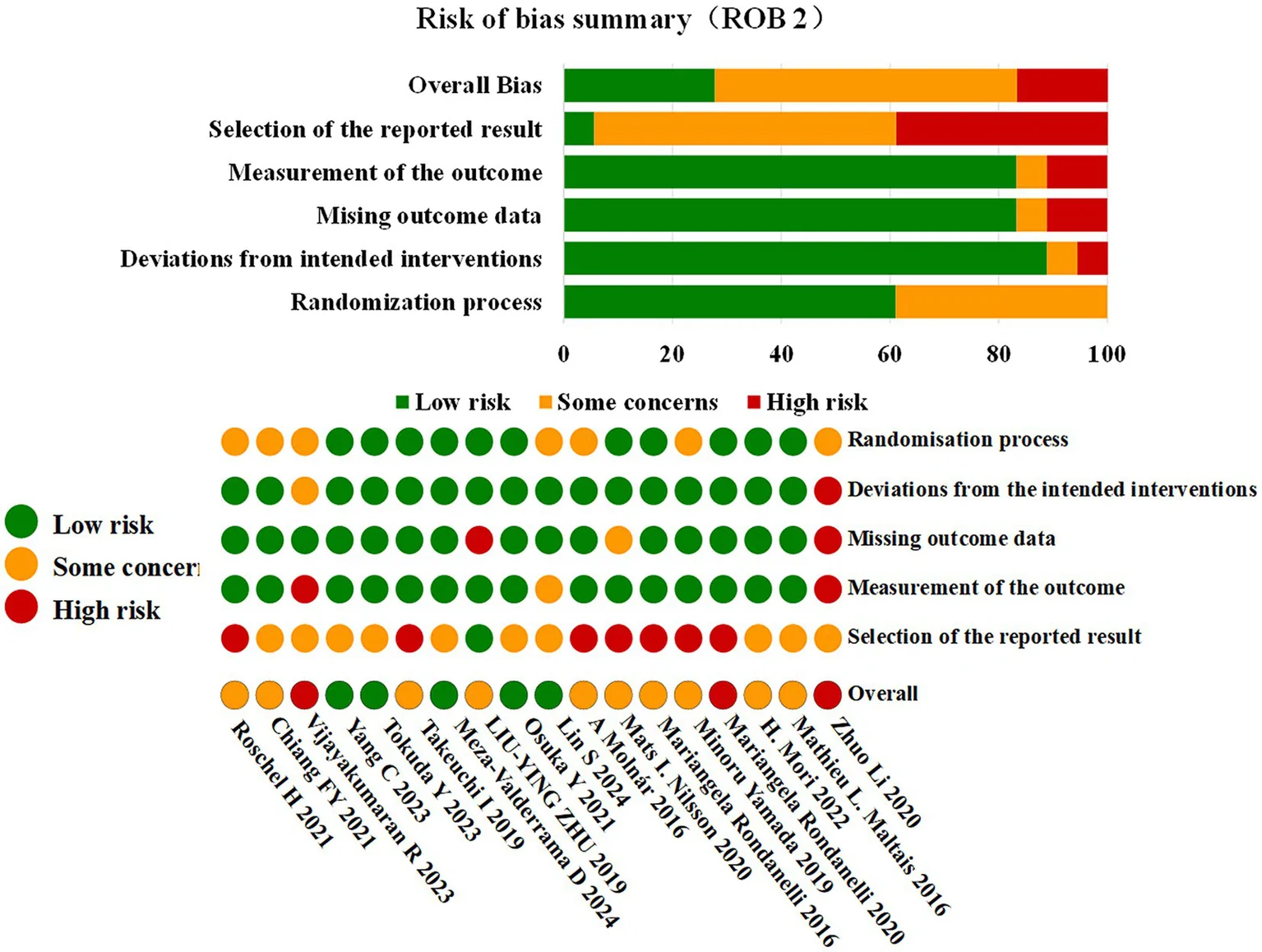
Risk-of-bias assessment of the included randomized controlled trials.

Overall, five studies received a low risk of bias rating, 10 studies raised some concerns, and three studies were judged to be high risk. The main sources of bias were selective reporting, missing outcome data, and outcome measurement. Figure 2 displays the detailed risk-of-bias assessment.

### 3.4 Pairwise meta-analysis

Sixteen studies comprising 1,149 participants reported grip strength. The pooled estimate showed moderate heterogeneity and indicated that adding protein or protein-related supplementation to resistance or functional exercise significantly improved grip strength compared with exercise plus placebo or exercise alone (SMD = 0.39, 95% CI: 0.20 to 0.58, *p* < 0.0001; *I*^2^ = 50%). Ten studies involving 625 participants reported ASMI. The pooled estimate exhibited no statistical heterogeneity and demonstrated a small but significant improvement in ASMI, favoring supplementation added to exercise-based interventions (SMD = 0.18, 95% CI: 0.00 to 0.35, *p* = 0.04; *I*^2^ = 0%). Four studies, contributing seven eligible comparisons and involving 457 participants, reported TUG performance. The pooled estimate revealed substantial heterogeneity, and the overall effect was not statistically significant (SMD = −0.39, 95% CI: −0.96 to 0.18, *p* = 0.18; *I*^2^ = 88%). Because lower TUG values indicate better physical performance, negative SMD values favored the intervention.

Subgroup analyses were conducted to explore potential sources of heterogeneity. For TUG performance, heterogeneity decreased after stratification by intervention duration and diagnostic criteria, suggesting that these factors may partly explain between-study heterogeneity. Interventions lasting less than 12 weeks showed a significant improvement in TUG performance, whereas those lasting 12 weeks or longer did not. However, this subgroup finding warrants cautious interpretation because the number of studies was small and individual studies strongly influenced the pooled estimate. Additional subgroup analyses based on exercise intensity and population status did not substantially reduce heterogeneity or reveal consistent between-subgroup differences. The pairwise meta-analysis results are presented in Supplementary Figures S1–S3.

### 3.5 Network meta-analysis

#### 3.5.1 Evidence network

**Figure 3:**
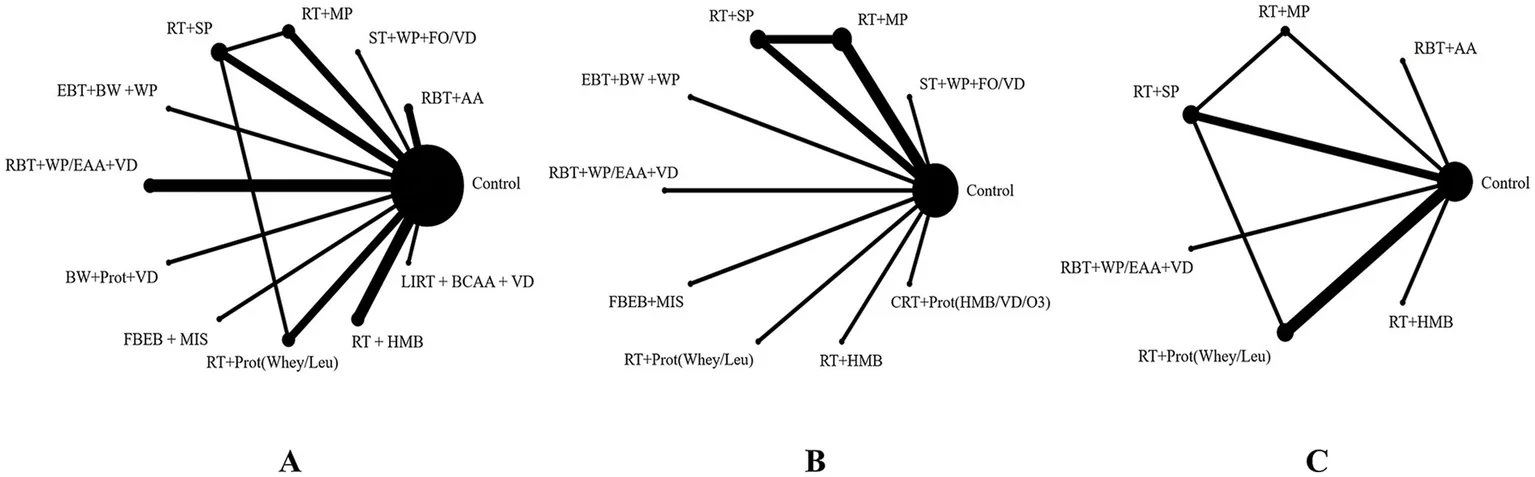
Network plots for grip strength, appendicular skeletal muscle mass index, and timed up-and-go test. Panel **A** shows the evidence network for grip strength, Panel **B** for appendicular skeletal muscle mass index (ASMI), and Panel **C** for timed up-and-go test (TUG). Each node represents an exercise-based protein or protein-related supplementation strategy. Node size is proportional to the sample size, and line thickness is proportional to the number of direct comparisons. The control node represents the same or comparable exercise program combined with placebo, no additional supplementation, or exercise alone. RT, resistance training; ST, strength training; EBT, elastic-band training; BW, body-weight exercise; RBT, resistance/balance/gait training; WP, whey protein; FO, fish oil; VD, vitamin D; EAA, essential amino acids; HMB, β-hydroxy-β-methylbutyrate; BCAA, branched-chain amino acids; MP, milk protein; SP, soy protein; MIS, multi-ingredient supplementation; Prot(Whey/Leu), whey protein or leucine-enriched protein. The network geometry represents the available direct and indirect comparisons among interventions. Comparisons supported by limited evidence should be interpreted cautiously due to the sparse structure of the evidence network.

Evidence networks were constructed for grip strength, ASMI, and TUG based on available direct comparisons between exercise-based protein or protein-related supplementation strategies and exercise-based comparators. Across all outcomes, the network included 12 exercise-based protein or protein-related supplementation strategies and one exercise-based comparator node. The number of available nodes differed by outcome. Figure 3 displays the network plots for each outcome.

Where closed loops were available, global inconsistency tests and node-splitting analyses detected no statistically significant inconsistency (*p* > 0.05). Accordingly, consistency models were used for the network meta-analyses. Nevertheless, given the sparse network structure and the limited number of trials supporting several intervention nodes, the absence of statistically significant inconsistency should not be interpreted as definitive evidence of full consistency.

The distributions of potential effect modifiers were examined across intervention comparisons to assess the plausibility of the transitivity assumption. Overall, included comparisons showed generally comparable participant characteristics regarding age range, sex distribution, sarcopenia diagnostic criteria, and baseline population status. Most exercise interventions consisted of resistance-based or functional exercise programs with similar intervention durations, although some variations were observed in exercise intensity, supplementation composition, and combined micronutrient strategies. These variations were considered potential sources of uncertainty when interpreting the network estimates. Detailed distributions of these characteristics across intervention nodes are presented in Supplementary Table S5.

#### 3.5.2 Grip strength

Given the very low certainty of evidence across all outcomes, the following network estimates should be interpreted as exploratory associations rather than confirmatory evidence.

Sixteen studies comprising 1,149 participants reported grip strength. Compared with the control condition, RT + HMB (SMD = 0.69, 95% CI: 0.18 to 1.20), RT + Prot (Whey/Leu) (SMD = 0.76, 95% CI: 0.16 to 1.35), and RBT + WP/EAA + VD (SMD = 0.73, 95% CI: 0.29 to 1.17) were associated with statistically significant estimates for grip strength improvement. The remaining intervention nodes did not demonstrate statistically significant differences relative to the control condition. Detailed pairwise comparisons are shown in Supplementary Figure S4.

**Figure 4:**
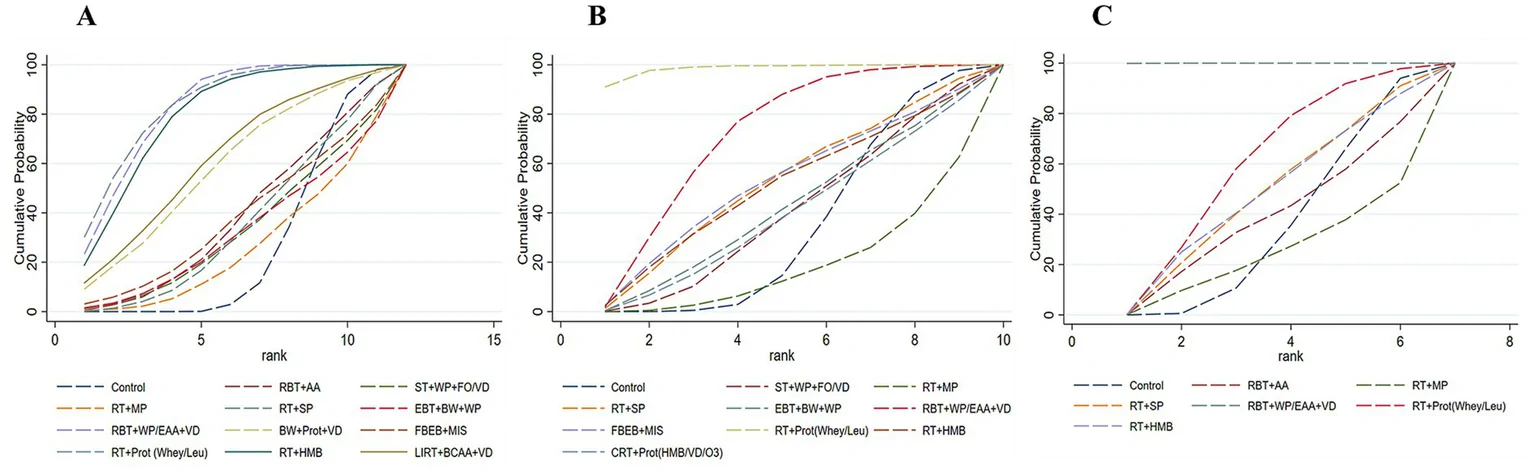
SUCRA ranking curves for grip strength, appendicular skeletal muscle mass index, and timed up-and-go test. Panel **A** shows the SUCRA ranking curves for grip strength, Panel **B** for appendicular skeletal muscle mass index (ASMI), and Panel **C** for timed up-and-go test (TUG). Higher SUCRA values indicate a higher probability of ranking among the more effective intervention strategies. Because several nodes were supported by limited direct evidence and small sample sizes, SUCRA rankings should be interpreted together with effect estimates, confidence intervals, network geometry, and sensitivity analyses. SUCRA, surface under the cumulative ranking curve; RT, resistance training; ST, strength training; EBT, elastic-band training; BW, body-weight exercise; RBT, resistance/balance/gait training; WP, whey protein; FO, fish oil; VD, vitamin D; EAA, essential amino acids; HMB, β-hydroxy-β-methylbutyrate; BCAA, branched-chain amino acids; MP, milk protein; SP, soy protein; MIS, multi-ingredient supplementation; Prot(Whey/Leu), whey protein or leucine-enriched protein. The SUCRA-based ranking probabilities represent relative estimates within the available evidence network. Given the sparse evidence network and very low certainty of evidence, these rankings should be interpreted as exploratory and should not be considered as establishing a definitive treatment hierarchy among supplementation strategies.

The SUCRA ranking (Figure 4A) suggested that RBT + AA (83.3%), RT + Prot (Whey/Leu) (83.1%), and RT + MP (80.5%) had relatively high probabilities of ranking among the most effective strategies for grip strength. However, several high-ranking nodes were supported by limited direct evidence or small sample sizes. Therefore, the SUCRA ranking for grip strength should be interpreted alongside the effect estimates, confidence intervals, network geometry, and sensitivity analyses, rather than as definitive evidence of superiority.

#### 3.5.3 Appendicular skeletal muscle mass index

Ten studies involving 625 participants reported ASMI. Compared with the control condition, RT + Prot (Whey/Leu) supplementation was associated with a statistically significant increase in ASMI estimates (SMD = 1.08, 95% CI: 0.41 to 1.75) compared with exercise alone. Other intervention strategies did not show statistically significant differences compared with the control condition. Detailed pairwise comparisons are presented in Supplementary Figure S5.

The SUCRA ranking (Figure 4B) indicated that RT + Prot (Whey/Leu) (98.7%) had the highest probability of being the most effective strategy for improving ASMI, followed by RBT + WP/EAA + VD (72.4%) and RT + SP (54%). These findings suggest that whey- or leucine-based supplementation, combined with resistance or functional exercise, may be particularly effective for improving muscle mass. Nevertheless, because several comparisons were based on limited evidence, the ranking results should be interpreted cautiously.

#### 3.5.4 Timed up-and-go test

Four studies, contributing seven eligible comparisons and involving 457 participants, reported TUG performance. Compared with the control condition, RBT + WP/EAA + VD was associated with significantly improved TUG performance (SMD = −2.01, 95% CI: −2.74 to −1.29). Because lower TUG values indicate better mobility, negative SMD values favored the intervention. However, the corresponding pairwise meta-analysis did not demonstrate a statistically significant overall effect on TUG performance, indicating that the observed network estimate should be interpreted cautiously. Detailed pairwise comparisons are provided in Supplementary Figure S6.

The SUCRA ranking (Figure 4C) indicated that RT + SP (77.4%), Control (65.5%), and RT + MP (62.4%) had relatively high probabilities of ranking high for TUG performance. However, the high ranking of the control condition and the small number of included studies indicate that the TUG network was sparse and unstable. Consequently, the ranking results for TUG should not be interpreted as reliable evidence that one supplementation strategy outperforms another. The observed benefit of RBT + WP/EAA + VD may reflect contributions from balance, gait, and task-specific functional training components rather than supplementation alone. Therefore, TUG should be considered an exploratory outcome requiring confirmation in future well-designed trials.

### 3.6 Sensitivity analysis

#### 3.6.1 Sensitivity analysis for pairwise Meta-analysis

Leave-one-out sensitivity analyses were performed for outcomes with substantial or moderate heterogeneity. For grip strength, heterogeneity decreased after excluding Rondanelli et al. (30) and Yang et al. (39) (*I*^2^ = 38 and 37%, respectively). The pooled effects remained statistically significant after either exclusion (SMD = 0.35, 95% CI: 0.17 to 0.53, *p* = 0.0002; SMD = 0.36, 95% CI: 0.19 to 0.53, *p* < 0.0001), indicating that the overall beneficial effect on grip strength was generally stable, although individual studies somewhat influenced the magnitude of the effect.

For TUG, heterogeneity decreased markedly after excluding Rondanelli et al. (28) (*I*^2^ = 0%). The pooled effect remained non-significant after exclusion (SMD = −0.11, 95% CI: −0.33 to 0.11, *p* = 0.32), indicating that the overall pairwise finding for TUG was not robust enough to support a clear additional benefit of supplementation added to exercise-based interventions. Sensitivity analysis results are shown in Supplementary Tables S6–S7.

#### 3.6.2 Sensitivity analysis for network Meta-analysis

To examine whether the network findings were influenced by low-frequency or small-sample intervention nodes, interventions that appeared only once in the original network were removed, and the models were refitted.

For grip strength, the statistical significance and relative ranking of several interventions changed after removing sparse nodes. In contrast, RT + Prot (Whey/Leu), RT + HMB, and RBT + WP/EAA + VD remained among the more favorable strategies, although the ranking order shifted. These findings suggest that the grip-strength network was only partially sensitive to sparse evidence, and that the ranking results should be interpreted cautiously.

For ASMI, the relative position of RT + Prot (Whey/Leu) remained generally stable after node removal, supporting the robustness of its favorable effect on muscle mass. For TUG, the network remained limited by the small number of studies, and the ranking results remained unstable. Overall, sensitivity analyses suggested that the ASMI findings were relatively more stable than the grip-strength and TUG findings. Detailed results are provided in Supplementary Tables S8–S10.

### 3.7 Publication bias

**Figure 5:**
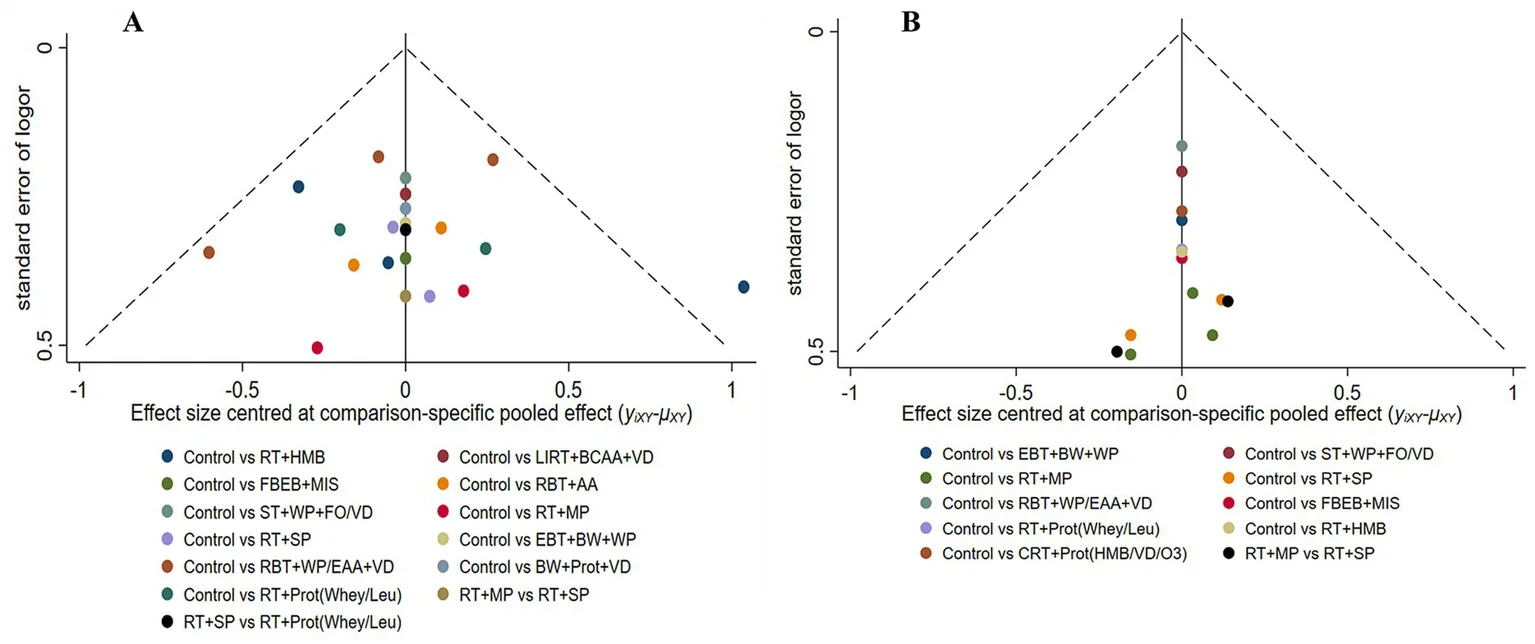
Comparison-adjusted funnel plots for grip strength and appendicular skeletal muscle mass index. Panel **A** shows the comparison-adjusted funnel plot for grip strength, and Panel **B** shows the comparison-adjusted funnel plot for appendicular skeletal muscle mass index (ASMI). Each point represents a comparison included in the network meta-analysis. The vertical line represents the comparison-specific pooled effect, and the dashed lines indicate the approximate 95% confidence limits. Asymmetry in the funnel plots may suggest possible publication bias, small-study effects, or heterogeneity across comparisons. ASMI, appendicular skeletal muscle mass index; RT, resistance training; ST, strength training; EBT, elastic-band training; BW, body-weight exercise; RBT, resistance/balance/gait training; WP, whey protein; FO, fish oil; VD, vitamin D; EAA, essential amino acids; HMB, β-hydroxy-β-methylbutyrate; BCAA, branched-chain amino acids; MP, milk protein; SP, soy protein; MIS, multi-ingredient supplementation; Prot(Whey/Leu), whey protein or leucine-enriched protein.

Comparison-adjusted funnel plots were generated for ASMI and grip strength, the outcomes with at least 10 included studies (Figure 5). The funnel plots showed asymmetrical distributions, with some points falling outside the funnel, suggesting possible publication bias or small-study effects. Because the number of studies for TUG was limited, a funnel plot for this outcome was deemed insufficiently informative.

### 3.8 Certainty of evidence

The GRADE assessment indicated that the certainty of evidence was very low for all three outcomes: grip strength, ASMI, and TUG. The main reasons for downgrading were risk of bias, inconsistency, potential publication bias or small-study effects, and imprecision due to sparse evidence and small sample sizes. Table 2 presents the detailed certainty-of-evidence assessment.

**Table 2:** GRADE certainty-of-evidence assessment.

Outcomes	Participants (studies)	Risk of bias	Inconsistency	Indirectness	Imprecision	Publication bias	Overall certainty of evidence
Grip strength	1,149 (16RCTs)	Serious	Seriousb	Not serious	Not serious	Serious^c^	⨁◯◯◯ Very low ^a, b, c^
ASMI	625 (10RCTs)	Serious	Not serious	Not serious	Not serious	Serious	⨁◯◯◯ Very low ^a, c^
TUG	457 (4 RCTs)	Serious^a^	Serious^b^	Not serious	serious^d^	Serious^c^	⨁◯◯◯ Very low ^a, b, c, d^

ASMI, Appendicular Skeletal Muscle Mass Index; TUG, Timed Up-and-Go Test; ᵃ Downgraded for risk of bias in the included studies; ᵇ Downgraded for substantial heterogeneity or instability across comparisons; ᶜ Downgraded for suspected publication bias or small-study effects; for outcomes with limited numbers of studies, publication bias could not be reliably excluded; ᵈ Downgraded for imprecision due to sparse evidence and small sample size.

## 4 Discussion

The present systematic review and network meta-analysis compared the incremental effects of different protein or protein-related supplementation strategies when added to resistance or functional exercise in older adults with sarcopenia or at high risk of sarcopenia. The pairwise meta-analysis demonstrated that supplementation added to resistance or functional exercise significantly improved grip strength and ASMI, whereas the overall effect on TUG performance was not statistically significant. The network meta-analysis further suggested outcome-specific patterns: RT + HMB, RT + Prot (Whey/Leu), and RBT + WP/EAA + VD were associated with statistically significant estimates for grip strength; RT + Prot (Whey/Leu) provided the clearest signal within the available network for ASMI; and RBT + WP/EAA + VD was associated with a potential improvement in TUG performance within the available network. These findings should be interpreted cautiously, however, because several intervention nodes were supported by limited evidence, the grip-strength rankings were sensitive to sparse nodes, and the certainty of evidence was very low for all outcomes. Given the very low certainty of evidence and sparse network structure, these findings should be considered hypothesis-generating.

The present findings should be interpreted as evidence regarding the potential additional contribution of supplementation to exercise-based interventions, rather than as evidence that one combined exercise–nutrition strategy is universally superior to others. Compared with previous network meta-analyses, the present study addressed a distinct clinical question. Liao et al. primarily investigated whether different protein supplementation strategies combined with resistance training resulted in differential effects on sarcopenia-related outcomes, whereas Zhao et al. evaluated broader exercise and nutrition strategies. In contrast, our study focused specifically on whether supplementation provides additional benefits beyond comparable resistance or functional exercise programs. This incremental perspective may provide more clinically relevant evidence because exercise is already considered a foundational component of sarcopenia management, and the practical decision faced by clinicians is whether additional supplementation is necessary.

The observed improvements in grip strength and ASMI align with the physiological rationale that protein or amino acid supplementation may potentiate resistance- or functional-exercise-induced adaptations. Resistance or functional exercise stimulates muscle protein synthesis and neuromuscular adaptation, while protein supplementation supplies the amino acid substrates required for muscle remodeling. Previous meta-analyses have shown that protein supplementation can augment resistance training-induced gains in muscle mass and strength. However, the magnitude of the benefit may vary with age, training status, total protein intake, and intervention characteristics (43, 44). In the current review, the effect size was larger for grip strength than for ASMI. This discrepancy suggests that strength-related adaptations may be more sensitive to short- and medium-term interventions than measurable changes in muscle mass. This pattern also aligns with the current diagnostic emphasis on muscle strength as an early and clinically meaningful marker of sarcopenia (1).

The network findings indicate that different supplementation strategies may not act uniformly across outcomes. For grip strength, strategies involving HMB, whey protein, leucine-enriched protein, or multi-ingredient supplementation combined with resistance or functional training showed significant benefits. These observations may be explained by their potential effects on muscle protein turnover. Whey protein is rapidly digested and rich in essential amino acids, particularly leucine, which plays an important role in stimulating postprandial muscle protein synthesis. Experimental evidence further suggests that older adults may require higher protein or leucine-rich protein doses to maximize post-exercise myofibrillar protein synthesis compared with younger adults (45, 46). HMB, a metabolite of leucine, may contribute by promoting muscle protein synthesis and attenuating proteolysis, although previous evidence in older adults remains mixed (47). Thus, the significant grip-strength effects observed for RT + HMB and RT + Prot (Whey/Leu) are biologically plausible but should still be considered preliminary, as small samples supported some nodes.

For ASMI, RT + Prot (Whey/Leu) exhibited the clearest advantage and the most stable ranking in sensitivity analysis. This finding suggests that whey protein- or leucine-based supplementation may be particularly relevant when the primary therapeutic goal is to preserve or increase muscle mass. However, the small pooled effect from the pairwise meta-analysis indicates that the absolute gain in ASMI may be modest. One possible explanation is that changes in muscle mass require sufficient intervention duration, training progression, energy intake, and adherence. In contrast, the included trials varied substantially in exercise intensity, supplementation dose, and baseline nutritional status. Another explanation involves anabolic resistance: older adults with sarcopenia may show a blunted muscle protein synthetic response to exercise and amino acid intake, requiring more targeted nutritional and training stimuli. Therefore, future trials should report not only the type of supplement but also the dose, timing, total daily protein intake, adherence, and progression of the exercise program.

The findings for TUG warrant particularly cautious interpretation. Although RBT + WP/EAA + VD was associated with improved TUG performance in the network meta-analysis, the pairwise meta-analysis showed no overall significant effect, and the TUG network comprised only 4 studies. TUG is a complex functional outcome involving lower-limb strength, balance, gait control, turning ability, and coordination. Accordingly, improvements in muscle mass or isolated grip strength may not directly translate into better mobility unless the exercise program includes balance, gait, and task-specific functional components. The apparent advantage of RBT + WP/EAA + VD may partly reflect the inclusion of resistance, balance, and gait training rather than the nutritional component alone. Consequently, nutritional supplementation is unlikely to improve mobility independently; instead, mobility-oriented benefits are likely to require an integrated intervention that combines adequate protein or amino acid intake with functional exercise components.

The interpretation of SUCRA rankings deserves particular caution. Although rankings are useful for summarizing the relative likelihood that each intervention is among the most effective strategies, they can be misleading when evidence networks are sparse, sample sizes are small, or confidence intervals are wide. In the present study, grip-strength rankings changed after removing low-frequency nodes, indicating that the ranking hierarchy was partly driven by limited direct evidence. The changes in intervention rankings after sensitivity analyses indicate that relative treatment hierarchies derived from sparse evidence networks should be interpreted cautiously. Similarly, the relatively high ranking of the control condition for TUG suggests instability rather than true superiority. Therefore, the most defensible conclusion is not that a single strategy is universally optimal, but that different supplementation-exercise combinations may show outcome-specific advantages. HMB-, whey protein-, or leucine-based strategies may be more relevant for muscle strength and muscle mass, whereas mobility improvement may depend more heavily on functional training components.

The present investigation has several limitations. First, the included trials differed in diagnostic criteria, participant status, intervention duration, exercise intensity, supplementation type, supplementation dose, and comparator design. These differences may have introduced clinical heterogeneity. Regarding the transitivity assumption, potential effect modifiers were assessed qualitatively; however, the limited number of studies within individual intervention nodes prevented formal evaluation using network meta-regression. Therefore, residual imbalance in intervention characteristics cannot be completely excluded. Second, several intervention nodes were supported by single or small trials, limiting the stability of network estimates and rankings. Third, some included studies had concerns regarding risk of bias, and the comparison-adjusted funnel plot suggested possible publication bias or small-study effects. Fourth, only a small number of studies reported TUG, limiting the ability to draw firm conclusions regarding physical performance. Although TUG provides clinically relevant information regarding functional mobility, it should not be considered equivalent to gait speed in sarcopenia assessment. Although gait speed was reported in 11 eligible trials, the available evidence network showed limited connectivity and potential concerns regarding network consistency, which limited our ability to include gait speed as a network meta-analysis outcome. Future randomized controlled trials should incorporate standardized physical performance outcomes, including gait speed and TUG, to facilitate more comprehensive evaluation of exercise–nutrition interventions for sarcopenia. Fifth, dose–response relationships according to protein dose, timing of supplementation intake, and adherence to exercise and supplementation protocols could not be adequately explored because these factors were inconsistently reported across the included trials. Future studies with standardized reporting of supplementation dosage, administration timing, and adherence measures are needed to clarify their potential influence on intervention effects. Finally, sex-specific effects could not be fully investigated. Due to insufficient sex-specific reporting and limited statistical power, the potential influence of sex on intervention effects could not be adequately explored and should be examined in future studies.

Future randomized controlled trials should adopt standardized sarcopenia diagnostic criteria, use adequately powered multi-arm designs, and provide detailed reporting of both exercise and nutritional intervention characteristics. In particular, future studies should report exercise type, intensity, progression, adherence, protein source, dose, timing of intake, total daily protein intake, and baseline nutritional status, as these factors may modify the response to exercise-based sarcopenia interventions (48). Such reporting would help clarify whether the observed benefits are attributable to specific supplementation strategies, exercise components, or their combined effects.

## 5 Conclusion

Protein or protein-related supplementation combined with resistance or functional exercise may provide additional benefits for grip strength and ASMI in older adults with sarcopenia or at high risk of sarcopenia. In contrast, evidence for improving TUG performance remains limited. The available network evidence suggests possible outcome-specific patterns: HMB-, whey protein-, or leucine-based strategies appeared more promising for muscle strength and muscle mass, whereas mobility-related improvements may depend more strongly on balance, gait, and task-specific functional exercise components. However, because the evidence network was sparse, several rankings were unstable, and the certainty of evidence was very low, no single supplementation strategy can be considered definitively superior. Future well-designed trials should standardize sarcopenia diagnostic criteria and provide detailed reporting of supplement dose, timing, total protein intake, exercise progression, adherence, and baseline nutritional status.

## Data Availability

The original contributions presented in the study are included in the article/Supplementary material, further inquiries can be directed to the corresponding author/s.
