# Supplementary material for: Comparative effectiveness of protein or protein-related supplementation combined with resistance or functional exercise for sarcopenia in older adults: a systematic review and network meta-analysis
Source: Front Nutr. 2026 Aug 25;13:1892302. doi: 10.3389/fnut.2026.1892302 (PMC13549823; doi:10.3389/fnut.2026.1892302)
Supplement: Supplementary file 1 [file Supplementary_file_1.DOCX]

Supplementary Material

# Supplementary Figures and Tables

## Table S1. Inclusion and exclusion criteria

| **Items** | **Inclusion criteria** | **Exclusion criteria** |
| --- | --- | --- |
| P (Population) | Participants aged 60 years or older with confirmed sarcopenia or at high risk of sarcopenia. Sarcopenia was diagnosed using recognized criteria, such as AWGS or EWGSOP criteria, or defined as high risk based on indicators such as FFMI, grip strength, SPPB, or SMI. | Participants with major comorbidities are likely to independently influence muscle-related outcomes; participants diagnosed only with “possible sarcopenia” or “pre-sarcopenia” without sufficient diagnostic information; studies using investigator-defined criteria without clear diagnostic documentation. |
| I (Intervention) | Resistance or function-oriented exercise combined with protein or protein-related supplementation. Function-oriented exercise was eligible when it included resistance, strengthening, balance, gait, chair-based, elastic-band, or body-weight components and when the exercise program was the same or comparable between groups. Protein-related supplementation included whey protein, milk protein, soy protein, leucine, essential amino acids, branched-chain amino acids, HMB, or multi-ingredient supplements containing protein- or amino-acid-related components. | Studies involving aerobic exercise only, non-exercise interventions, or exercise programs that differed substantially between intervention and control groups. Studies that only provided dietary advice to increase protein intake without quantifiable supplemental doses were also excluded. |
| C (Comparator) | The same or comparable resistance- or function-oriented exercise program combined with placebo, no additional supplementation, or exercise alone. | Studies without a parallel control design; studies in which the exercise program differed substantially between groups; studies with major baseline differences in protein intake, total energy intake, or physical activity level without statistical adjustment. |
| O (Outcome) | At least one of the following outcomes: appendicular skeletal muscle mass index (ASMI), grip strength, or timed up-and-go test (TUG). | Studies without extractable data for ASMI, grip strength, or TUG. |
| S (Study design) | Individual or cluster randomized controlled trials. | Non-randomized studies, uncontrolled pre-post studies, case reports, descriptive studies, reviews, dissertations, conference abstracts, or protocols without outcome data. |
| T (Publication and search period) | PubMed, Embase, the Cochrane Library, Web of Science, CNKI, and SinoMed were searched from database inception to March 16, 2026, without language restrictions. | Studies for which the full text or necessary outcome data could not be obtained. |

## Table S2. Intervention node classification for grip strength

| **Node** | **Full name** | **Nutritional component** | **Exercise component** | **Included studies/comparisons** | **Reason for grouping** |
| --- | --- | --- | --- | --- | --- |
| Control | Exercise-based comparator plus placebo/no additional supplementation | Placebo or no additional supplementation | Same or comparable resistance- or function-oriented exercise program | Li 2021; Mori 2022; Rondanelli 2020; Yamada 2019; Rondanelli 2016; Nilsson 2020; Molnár 2016; Lin 2024; Osuka 2021; Meza-Valderrama 2024; Takeuchi 2019; Tokuda 2023; Yang 2023; Vijayakumaran 2023; Chiang 2021; Roschel 2021 | Common exercise-based comparator |
| ST+WP+FO/VD | Strength training plus whey protein, fish oil, and vitamin D | Whey protein + fish oil + vitamin D | Strength training | Li 2021 | Multi-nutrient supplementation with strength training |
| EBT + BW + WP | Elastic-band training plus body-weight exercise and whey protein | Whey protein | Elastic-band training + body-weight exercise | Mori 2022 | Combined resistance modality with whey protein |
| RBT+WP/EAA+VD | Resistance/balance/gait training plus whey protein/essential amino acids and vitamin D | Whey protein or essential amino acids + vitamin D | Resistance/balance/gait training | Rondanelli 2020; Rondanelli 2016; Molnár 2016 | Multi-component nutrition plus functional training |
| BW + Prot + VD | Body-weight resistance exercise plus protein and vitamin D supplementation | Protein + vitamin D | Body-weight resistance exercise | Yamada 2019 | Body-weight exercise with protein/vitamin D supplementation |
| FBEB+MIS | Full-body elastic-band exercise plus multi-ingredient supplementation | Multi-ingredient supplementation | Full-body elastic-band exercise | Nilsson 2020 | Elastic-band exercise with multi-ingredient supplementation |
| RT+Prot(Whey/Leu) | Resistance training plus whey or leucine-enriched protein | Whey protein or leucine-enriched protein | Resistance training | Lin 2024; Roschel 2021 | Leucine-rich protein strategy |
| RT+HMB | Resistance training plus HMB/Ca-HMB | HMB or Ca-HMB | Resistance exercise | Osuka 2021; Meza-Valderrama 2024; Yang 2023 | Same main anabolic metabolite |
| LIRT+BCAA+VD | Low-intensity resistance training plus BCAA and vitamin D | Branched-chain amino acids + vitamin D | Low-intensity resistance training | Takeuchi 2019 | Low-intensity resistance training with BCAA/vitamin D |
| RBT+AA | Resistance/balance/gait training plus amino acids | Amino acids, such as EAA or leucine | Resistance/balance/gait training | Tokuda 2023; Roschel 2021 | Amino acid supplementation with functional training |
| RT+MP | Resistance training plus milk protein | Milk protein | Resistance training | Vijayakumaran 2023; Chiang 2021 | Dairy-based protein supplementation |
| RT+SP | Resistance training plus soy protein | Soy protein | Resistance training | Chiang 2021; Roschel 2021 | Plant-based protein supplementation |

Note on node classification: Intervention nodes were classified primarily according to the dominant protein or protein-related supplementation component and, where clinically relevant, the accompanying exercise modality. Exercise components were required to be the same or comparable between intervention and comparator groups within each trial. Therefore, the network estimates represent the additional effect of protein or protein-related supplementation within an exercise-based intervention framework, rather than the isolated effect of exercise alone. For multi-arm or multiple-comparison trials, the same study may appear in more than one intervention node when it contributed more than one eligible comparison.

## Table S3. Intervention node classification for appendicular skeletal muscle mass index (ASMI)

| **Node** | **Full name** | **Nutritional component** | **Exercise component** | **Included studies/comparisons** | **Reason for grouping** |
| --- | --- | --- | --- | --- | --- |
| Control | Exercise-based comparator plus placebo/no additional supplementation | Placebo or no additional supplementation | Same or comparable resistance- or function-oriented exercise program | Li 2021; Maltais 2016; Mori 2022; Rondanelli 2020; Nilsson 2020; Lin 2024; Zhu 2019; Yang 2023; Vijayakumaran 2023; Chiang 2021 | Common exercise-based comparator |
| ST+WP+FO/VD | Strength training plus whey protein, fish oil, and vitamin D | Whey protein + fish oil + vitamin D | Strength training | Li 2021 | Multi-nutrient supplementation with strength training |
| RT+MP | Resistance training plus milk protein | Milk protein | Resistance training | Maltais 2016; Vijayakumaran 2023; Chiang 2021 | Dairy-based protein supplementation |
| RT+SP | Resistance training plus soy protein | Soy protein | Resistance training | Maltais 2016; Chiang 2021 | Plant-based protein supplementation |
| EBT+BW+WP | Elastic-band training plus body-weight exercise and whey protein | Whey protein | Elastic-band training + body-weight exercise | Mori 2022 | Combined resistance modality with whey protein |
| RBT+WP/EAA+VD | Resistance/balance/gait training plus whey protein/essential amino acids and vitamin D | Whey protein or essential amino acids + vitamin D | Resistance/balance/gait training | Rondanelli 2020 | Multi-component nutrition plus functional training |
| FBEB+MIS | Full-body elastic-band exercise plus multi-ingredient supplementation | Multi-ingredient supplementation | Full-body elastic-band exercise | Nilsson 2020 | Elastic-band exercise with multi-ingredient supplementation |
| RT+Prot(Whey/Leu) | Resistance training plus whey or leucine-enriched protein | Whey protein or leucine-enriched protein | Resistance training | Lin 2024 | Leucine-rich protein strategy |
| CRT+Prot(HMB/VD/O3) | Chair-based resistance training plus protein-related supplementation containing HMB, vitamin D, and omega-3 fatty acids | HMB + vitamin D + omega-3 fatty acids | Chair-based resistance training | Zhu 2019 | Seated resistance training with multi-nutrient supplementation |
| RT+HMB | Resistance training plus HMB/Ca-HMB | HMB or Ca-HMB | Resistance exercise | Yang 2023 | Same main anabolic metabolite |

Note on node classification: Intervention nodes were classified primarily according to the dominant protein or protein-related supplementation component and, where clinically relevant, the accompanying exercise modality. Exercise components were required to be the same or comparable between intervention and comparator groups within each trial. Therefore, the network estimates represent the additional effect of protein or protein-related supplementation within an exercise-based intervention framework, rather than the isolated effect of exercise alone. For multi-arm or multiple-comparison trials, the same study may appear in more than one intervention node when it contributed more than one eligible comparison.

## Table S4. Intervention node classification for the timed up-and-go test (TUG)

| **Node** | **Full name** | **Nutritional component** | **Exercise component** | **Included studies/comparisons** | **Reason for grouping** |
| --- | --- | --- | --- | --- | --- |
| Control | Exercise-based comparator plus placebo/no additional supplementation | Placebo or no additional supplementation | Same or comparable resistance- or function-oriented exercise program | Maltais 2016; Rondanelli 2020; Osuka 2021; Roschel 2021 | Common exercise-based comparator |
| RT+MP | Resistance training plus milk protein | Milk protein | Resistance training | Maltais 2016 | Dairy-based protein supplementation |
| RT+SP | Resistance training plus soy protein | Soy protein | Resistance training | Maltais 2016; Roschel 2021 | Plant-based protein supplementation |
| RBT+WP/EAA+VD | Resistance/balance/gait training plus whey protein/essential amino acids and vitamin D | Whey protein or essential amino acids + vitamin D | Resistance/balance/gait training | Rondanelli 2020 | Multi-component nutrition plus functional training |
| RT+HMB | Resistance training plus HMB/Ca-HMB | HMB or Ca-HMB | Resistance exercise | Osuka 2021 | Same main anabolic metabolite |
| RBT+AA | Resistance/balance/gait training plus amino acids | Amino acids, such as EAA or leucine | Resistance/balance/gait training | Roschel 2021 | Amino acid supplementation with functional training |
| RT+Prot(Whey/Leu) | Resistance training plus whey or leucine-enriched protein | Whey protein or leucine-enriched protein | Resistance training | Roschel 2021 | Leucine-rich protein strategy |

Note on node classification: Intervention nodes were classified primarily according to the dominant protein or protein-related supplementation component and, where clinically relevant, the accompanying exercise modality. Exercise components were required to be the same or comparable between intervention and comparator groups within each trial. Therefore, the network estimates represent the additional effect of protein or protein-related supplementation within an exercise-based intervention framework, rather than the isolated effect of exercise alone. For multi-arm or multiple-comparison trials, the same study may appear in more than one intervention node when it contributed more than one eligible comparison.

## Table S5. Distribution of potential effect modifiers across intervention nodes for qualitative assessment of transitivity

| **Intervention node** | **Age range (years)** | **Sex distribution** | **Population status / diagnostic criteria** | **Exercise modality** | **Exercise intensity** | **Intervention duration** | **Supplementation characteristics** |
| --- | --- | --- | --- | --- | --- | --- | --- |
| Exercise alone (control) | 64–85 | Male and female participants | Confirmed sarcopenia or at risk; AWGS/EWGSOP/other criteria | Resistance training, balance training, functional exercise, or usual exercise program | Low to high | 8–48 weeks | No additional protein or protein-related supplementation |
| RT + milk protein (RT+MP) | 68–84 | Mainly male participants; some mixed-sex populations | Confirmed sarcopenia or at risk; AWGS/EWGSOP criteria | Resistance training | High | 12–16 weeks | Milk protein supplementation |
| RT + soy protein (RT+SP) | 64–85 | Male and female participants | Confirmed sarcopenia or at risk; AWGS/other criteria | Resistance training | High | 12–16 weeks | Soy protein supplementation |
| RT + whey protein / leucine-enriched protein (RT + Prot [Whey/Leu]) | 72–78 | Male and female participants | Confirmed sarcopenia or at risk; AWGS/other criteria | Resistance training | High | 12–48 weeks | Whey protein or leucine-enriched protein supplementation |
| RT + HMB (RT+HMB) | 73–82 | Mainly female participants; mixed-sex populations | Confirmed sarcopenia or at risk; AWGS/EWGSOP criteria | Resistance training | Low to high | 12 weeks | HMB or calcium-HMB supplementation |
| RBT + WP/EAA + VD | 66–84 | Male and female participants | Confirmed sarcopenia; AWGS/EWGSOP criteria | Resistance training combined with balance/gait training | Low to high | 12 weeks | Whey protein or essential amino acids combined with vitamin D |
| ST + WP + FO/VD | 71–74 | Male and female participants | Confirmed sarcopenia; AWGS criteria | Strength training | High | 12 weeks | Whey protein combined with fish oil and vitamin D |
| EBT + BW + WP | 77–78 | Male and female participants | Confirmed sarcopenia; AWGS criteria | Elastic band training plus body-weight exercise | High | 48 weeks | Whey protein supplementation |
| BW + Prot + VD | 85 years approximately | Male and female participants | Confirmed sarcopenia or at risk; AWGS criteria | Body-weight exercise | Low | 12 weeks | Protein supplementation combined with vitamin D |
| FBEB + MIS | 74–77 | Male participants | Confirmed sarcopenia; EWGSOP criteria | Full-body elastic band exercise | Low | 12 weeks | Multi-ingredient supplementation containing protein-related components |
| CRT + Prot (HMB/VD/O3) | 75 years approximately | Male and female participants | Confirmed sarcopenia; AWGS criteria | Chair-based resistance training | Low | 12 weeks | Protein-related supplementation containing HMB, vitamin D, and omega-3 fatty acids |
| LIRT + BCAA + VD | 79–81 | Male and female participants | Confirmed sarcopenia; AWGS criteria | Low-intensity resistance training | Low | 8 weeks | Branched-chain amino acids plus vitamin D |
| RBT + AA | 72 years approximately | Male participants | At risk of sarcopenia; other diagnostic criteria | Resistance/balance/gait training | High | 16–24 weeks | Amino acids, including essential amino acids or leucine |

Note: RT, resistance training; RBT, resistance/balance/gait training; ST, strength training; EBT, elastic band training; BW, body-weight exercise; FBEB, full-body elastic band exercise; CRT, chair-based resistance training; LIRT, low-intensity resistance training; WP, whey protein; EAA, essential amino acids; AA, amino acids; MP, milk protein; SP, soy protein; HMB, β-hydroxy-β-methylbutyrate; VD, vitamin D; FO, fish oil; O3, omega-3 fatty acids; MIS, multi-ingredient supplementation. Values are summarized at the intervention-node level based on the characteristics of included randomized controlled trials. This table was used to support qualitative assessment of the transitivity assumption in the network meta-analysis.

## Table S6. Leave-one-comparison-out sensitivity analysis for grip strength in pairwise meta-analysis

| **Excluded study/comparison** | **Pooled SMD (95% CI)** | **P (Total)** | **I² (%)** | **P (I^2^)** |
| --- | --- | --- | --- | --- |
| Original merge (unfiltered) | 0.39 (0.20, 0.58) | ＜0.0001 | 50% | 0.007 |
| Zhuo Li 2020 | 0.42 (0.23, 0.61) | ＜0.0001 | 47% | 0.01 |
| H. Mori 2022 | 0.41 (0.22, 0.61) | ＜0.0001 | 50% | 0.009 |
| Mariangela Rondanelli 2020 | 0.36 (0.17, 0.56) | 0.0003 | 49% | 0.01 |
| Minoru Yamada 2019 | 0.39 (0.19, 0.59) | 0.0001 | 52% | 0.005 |
| Mariangela Rondanelli 2016 | 0.35 (0.17, 0.53) | 0.0002 | 38% | 0.05 |
| Mats I. Nilsson 2020 | 0.41 (0.21, 0.60) | ＜0.0001 | 51% | 0.007 |
| A Molnár 2016 | 0.40 (0.20, 0.60) | ＜0.0001 | 52% | 0.006 |
| Lin S 2024 | 0.36 (0.17, 0.55) | 0.0002 | 48% | 0.01 |
| Osuka Y 2021 | 0.40 (0.20, 0.60) | ＜0.0001 | 52% | 0.006 |
| Meza-Valderrama D 2024 | 0.38 (0.19, 0.58) | 0.0001 | 52% | 0.005 |
| Takeuchi I 2019 | 0.39 (0.18, 0.59) | 0.0002 | 53% | 0.005 |
| Tokuda Y 2023 | 0.41 (0.22, 0.60) | ＜0.0001 | 50% | 0.008 |
| Yang C 2023 | 0.36 (0.19, 0.53) | ＜0.0001 | 37% | 0.06 |
| Vijayakumaran R 2023 | 0.41 (0.22, 0.60) | ＜0.0001 | 49% | 0.010 |
| Chiang FY 2021 | 0.40 (0.21, 0.60) | ＜0.0001 | 52% | 0.006 |
| Chiang FY 2021 | 0.40 (0.21, 0.60) | ＜0.0001 | 52% | 0.006 |
| Roschel H 2021 | 0.40 (0.20, 0.60) | ＜0.0001 | 52% | 0.006 |
| Roschel H 2021 | 0.38 (0.18, 0.58) | 0.0002 | 52% | 0.005 |
| Roschel H 2021 | 0.41 (0.22, 0.61) | ＜0.0001 | 49% | 0.009 |

Note: For multi-arm trials contributing more than one eligible comparison, sensitivity analyses were conducted at the comparison level. Therefore, the same study may appear more than once when it contributed multiple eligible intervention-control comparisons.

## Table S7. Leave-one-comparison-out sensitivity analysis for the timed up-and-go test in a pairwise meta-analysis

| **Excluded study/comparison** | **Pooled SMD (95% CI)** | **P (Total)** | **I² (%)** | **P (I^2^)** |
| --- | --- | --- | --- | --- |
| Original merge (unfiltered) | -0.39 (-0.96, 0.18) | 0.18 | 88% | ＜0.00001 |
| Mathieu L. Maltais 2016 | -0.41 (-1.03, 0.20) | 0.19 | 89% | ＜0.00001 |
| Mathieu L. Maltais 2016 | -0.32 (-0.93, 0.29) | 0.30 | 89% | ＜0.00001 |
| Mariangela Rondanelli 2020 | -0.11 (-0.33, 0.11) | 0.32 | 0% | 0.57 |
| Osuka Y 2021 | -0.42 (-1.08, 0.23) | 0.21 | 89% | ＜0.00001 |
| Roschel H 2021 | -0.44 (-1.07, 0.19) | 0.17 | 89% | ＜0.00001 |
| Roschel H 2021 | -0.43 (-1.07, 0.21) | 0.19 | 89% | ＜0.00001 |
| Roschel H 2021 | -0.48 (-1.09, 0.13) | 0.12 | 88% | ＜0.00001 |
| Roschel H 2021 | -0.41 (-1.05, 0.24) | 0.22 | 89% | ＜0.00001 |
| Roschel H 2021 | -0.42 (-1.06, 0.22) | 0.20 | 89% | ＜0.00001 |

Note: For multi-arm trials contributing more than one eligible comparison, sensitivity analyses were conducted at the comparison level. Therefore, the same study may appear more than once when it contributed multiple eligible intervention-control comparisons.

## Table S8. Sensitivity analysis after removing sparse intervention nodes for grip strength in network meta-analysis

| **Remaining interventions** | **Before removal SMD (95%CI)** | **Before removal SUCRA** | **After removal SMD (95%CI)** | **After removal SUCRA** |
| --- | --- | --- | --- | --- |
| RBT+AA | 0.12 (-0.50, 0.75) | 83.3% | 0.12 (-0.50, 0.75) | 34.1% |
| RT+MP | -0.05 (-0.77, 0.67) | 80.5% | -0.05 (-0.77, 0.67) | 22.3% |
| RT+SP | 0.07 (-0.54, 0.68) | 62.6% | 0.07 (-0.54, 0.68) | 29.4% |
| RBT+WP/EAA+VD | 0.73 (0.29, 1.17) | 27.0% | 0.73 (0.29, 1.17) | 81.4% |
| RT+Prot(Whey/Leu) | 0.76 (0.16, 1.35) | 83.1% | 0.76 (0.16, 1.35) | 82.9% |
| RT+HMB | 0.69 (0.18, 1.20) | 58.4% | 0.69 (0.18, 1.20) | 77.7% |

Note: Sparse intervention nodes were defined as nodes supported by only one eligible study or a very small number of participants in the original network. The sensitivity analysis was conducted to examine whether network estimates and SUCRA rankings were influenced by low-frequency nodes. SMD values are shown relative to the exercise-based comparator unless otherwise specified.

## Table S9. Sensitivity analysis after removing sparse intervention nodes for appendicular skeletal muscle mass index (ASMI) in network meta-analysis

| **Remaining interventions** | **Before removal SMD (95%CI)** | **Before removal SUCRA** | **After removal SMD (95%CI)** | **After removal SUCRA** |
| --- | --- | --- | --- | --- |
| RT+MP | -0.19 (-0.71, 0.33) | 18.8% | -0.19 (-0.71, 0.33) | 18.2% |
| RT+SP | 0.16 (-0.44, 0.76) | 53.5% | 0.16 (-0.44, 0.76) | 78.8% |

Note: Sparse intervention nodes were defined as nodes supported by only one eligible study or a very small number of participants in the original network. The sensitivity analysis was conducted to examine whether network estimates and SUCRA rankings were influenced by low-frequency nodes. SMD values are shown relative to the exercise-based comparator unless otherwise specified.

## Table S10. Sensitivity analysis after removing sparse intervention nodes for the timed up-and-go test (TUG) in network meta-analysis

| **Remaining interventions** | **Before removal SMD (95%CI)** | **Before removal SUCRA** | **After removal SMD (95%CI)** | **After removal SUCRA** |
| --- | --- | --- | --- | --- |
| RT+SP | 0.14 (-0.59, 0.86) | 77.4% | -0.06 (-0.79, 0.66) | 55.8% |
| RT+Prot(Whey/Leu) | 0.25 (-0.23, 0.71) | 52.5% | -0.23 (-0.64, 0.18) | 22.8% |

Note: Sparse intervention nodes were defined as nodes supported by only one eligible study or a very small number of participants in the original network. The sensitivity analysis was conducted to examine whether network estimates and SUCRA rankings were influenced by low-frequency nodes. SMD values are shown relative to the exercise-based comparator unless otherwise specified.

**Figure S1.** Forest plot for grip strength in pairwise meta-analysis.

**Figure S2.** Forest plot for appendicular skeletal muscle mass index (ASMI) in pairwise meta-analysis.

**Figure S3.** Forest plot for the timed up-and-go test (TUG) in pairwise meta-analysis.

**Figure S4.** League table for grip strength in network meta-analysis.

**Figure S5.** League table for appendicular skeletal muscle mass index (ASMI) in network meta-analysis.

**Figure S6.** League table for timed up-and-go test (TUG) in network meta-analysis.
